# Molecular Targets for the Treatment of Metastatic Colorectal Cancer

**DOI:** 10.3390/cancers12092350

**Published:** 2020-08-20

**Authors:** Romain Cohen, Thomas Pudlarz, Jean-François Delattre, Raphaël Colle, Thierry André

**Affiliations:** Department of Medical Oncology, Hôpital Saint-Antoine, Sorbonne Université, Assistance Publique-Hôpitaux de Paris (AP-HP), F-75012 Paris, France; thomas.pudlarz@gmail.com (T.P.); jfdelattre2509@hotmail.fr (J.-F.D.); raphael.colle@aphp.fr (R.C.); thierry.andre@aphp.fr (T.A.)

**Keywords:** colorectal cancer, *NTRK*, *HER2*, *BRAF*, microsatellite instability, ctDNA

## Abstract

Over the past years, colorectal cancer (CRC) was subtyped according to its molecular and genetic characteristics, allowing the development of therapeutic strategies, based on predictive biomarkers. Biomarkers such as microsatellite instability (MSI), *RAS* and *BRAF* mutations, *HER2* amplification or *NTRK* fusions represent major tools for personalized therapeutic strategies. Moreover, the routine implementation of molecular predictive tests provides new perspectives and challenges for the therapeutic management of CRC patients, such as liquid biopsies and the reintroduction of anti-EGFR monoclonal antibodies. In this review, we summarize the current landscape of targeted therapies for metastatic CRC patients, with a focus on new developments for EGFR blockade and emerging biomarkers (MSI, *HER2*, *NTRK*).

## 1. Introduction

Colorectal cancer (CRC) is the third most common cancer and the second leading cause of death worldwide [1]. Median overall survival (OS) in patients with previously untreated and unresectable advanced colorectal (CRC) cancer now ranges from 25 to 30 months, when combining molecular targeted therapies and chemotherapy [2].

Whereas treatment for localized CRC rely on surgery and chemotherapy, therapeutic strategies in metastatic setting were improved through the development of biomarkers for targeted therapies, such as EGFR (epithelial growth factor receptor) inhibitors, *BRAF* serine/threonine-protein kinase B-Raf) inhibitors, *HER2* (human epidermal growth factor receptor 2) inhibitors, immune checkpoint inhibitors, or *NTRK* (neurotrophic tyrosine receptor kinase) inhibitors. Predictive biomarkers for mCRC patients now include *RAS* (Kirsten rat sarcoma virus) and *BRAF* mutations, microsatellite instability (MSI) and mismatch repair deficiency (dMMR), *HER2* amplifications and *NTRK* fusions. The challenge is now to implement these innovations in daily practice.

In this review, we discuss current predictive biomarkers for mCRC patients and their associated targeted therapy, including emerging parameters for anti-EGFR agents, such as primary tumor sidedness and longitudinal follow-up using circulating tumor DNA (ctDNA), immune checkpoint inhibitors for MSI/dMMR tumors, new developments for *BRAF*^V600E^-mutated cancers, anti-HER2 treatments and *NTRK* inhibitors.

## 2. *RAS*/*RAF* Wild-Type Tumors and Anti-EGFR Agents

### 2.1. Predictive Factors for The Efficacy of Anti-EGFR Agents

**Figure 1:**
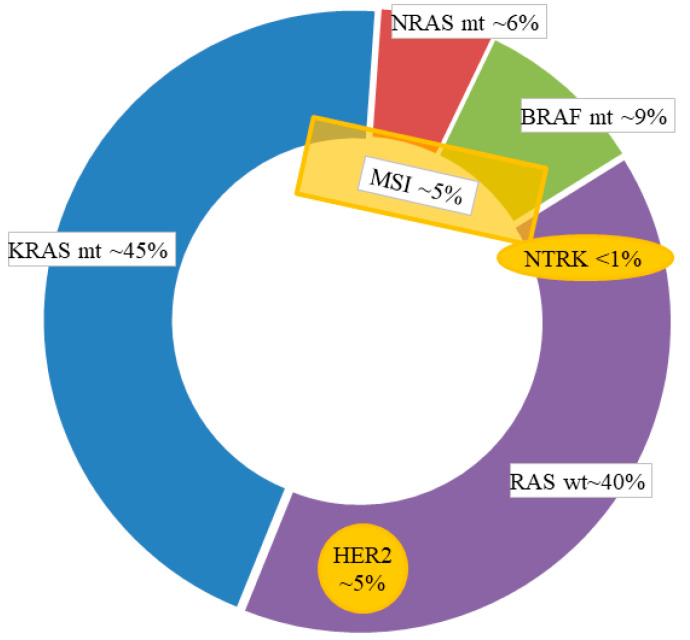
Molecular targets in metastatic colorectal cancer.

Activating mutations of *KRAS* and *NRAS* are predictive of resistance to anti-EGFR agents in CRC patients (see Figure 1) [3]. *KRAS* is mutated in 40 to 50% of CRC and *NRAS* in 4 to 8% [4]. Recommendations for *RAS* mutation testing include *KRAS* exons 2, 3, and 4 (codons 12, 13, 59, 61, 117, and 146) and *NRAS* exons 2, 3, and 4 (codons 12, 13, 59, 61, and 117) [5,6]. Patients with *KRAS*/*NRAS* wild type (WT) are eligible to treatments targeting the EGFR.

Other mechanisms of resistance are described. These might involve the EGFR itself with mutations of the EGFR ectodomain [7]. It can also be related to the overactivation of a protein downstream from the EGFR in the MAPK (mitogen-activated protein kinase) pathway, such as *BRAF* [8], although the *BRAF*^V600E^ hotspot mutation was not formally demonstrated as a factor of resistance to anti-EGFR agents (see below). The constitutional activation of the PI3K/Akt/mTOR pathway through *PIK3CA* exon 20 mutation or *PTEN* loss, could participate in resistance to monoclonal antibodies (mAbs) [9,10]. Furthermore, amplifications of *HER2*, *HER3*, or *MET* and *HER2* activating mutations seem associated with resistance to anti-EGFR therapy [11]. Recent reports brought to light the predictive value of the micro RNA miR-31-3p. Mir-31 plays a significant role in activating the *RAS* signaling pathway, and high miR-31-3p expression could be the witness of the tumor’s EGFR independency and subsequently to its resistance to anti-EGFR therapy. Several post hoc analysis of randomized trials showed the level of expression of miR-31-3p is a valid biomarker of anti-EGFR efficacy [12,13,14].

### 2.2. Current Management of Anti-EGFR Therapy

First, anti-EGFR mAbs do not improve outcomes as adjuvant treatment for resected stage III colon cancer [15]. Concerning patients with resectable mCRC, a warning signal concerning the use of anti-EGFR mAbs in the peri-operative setting was raised by the NEW EPOC trial. This study showed a detrimental effect of cetuximab, both on disease free survival (DFS) and overall survival (OS), when added to chemotherapy [16]. Although anti-EGFR mAbs should not be used as perioperative treatment for patients with resectable mCRC, these treatments could be useful as a converting therapy to shrink potentially resectable metastatic diseases [17].

Anti-EGFR mAbs (cetuximab and panitumumab) are associated with improved response rates, progression free survival (PFS), and OS in first-line setting, when combined with an oxaliplatin-based or irinotecan-based regimen, as well as in second or latter lines alone, or combined with cytotoxic agents [18,19,20,21,22,23,24,25,26,27,28,29]. Although the NORDIC VII and COIN studies did not show a significant effect of cetuximab in combination with an oxaliplatin-based regimen [30,31], recent data provided by the TAILOR trial demonstrate that it can be safely prescribed with FOLFOX for *RAS* WT mCRC patients [32]. There is no direct comparison between panitumumab and cetuximab, except for chemo-resistant patients with the ASPECCT trial that showed the non-inferiority of panitumumab in patients with chemotherapy-refractory *KRAS* WT (exon 2) mCRC [33].

### 2.3. Primary Tumor Sidedness

Primary tumor sidedness (PTS) seems to play a decisive role in the sensitivity to anti-EGFR mAbs. Indeed, there is growing evidence showing that the PTS is a prognostic factor in the *RAS* WT population, as well as a predictive factor of response to anti-EGFR mAbs [34]. A retrospective analysis of six randomized trials (CRYSTAL, FIRE-3, CALGB 80405, PRIME, PEAK, and 20050181) showed poorer outcomes (response rates, PFS, OS) for right-sided tumors, compared to left-sided tumors. This analysis also showed a predictive effect of PTS, with a greater effect of chemotherapy plus anti-EGFR mAbs, compared to chemotherapy ± bevacizumab in patients with left-sided tumors [35]. A recent retrospective analysis of the ARCAD database showed that the prognostic value of PTS was restricted to the *KRAS* WT population and confirmed the predictive value of PTS for cetuximab efficacy, with improved outcomes for left-sided mCRC patients [36]. On the contrary, patients with *RAS* WT right-sided mCRC seem to derive limited benefit from anti-EGFR treatments.

Although these results should be interpreted with caution due to their retrospective nature, they suggest that one should use anti-EGFR mAbs plus chemotherapy as the first line treatment only in patients with *KRAS*/*NRAS* WT and left-sided tumors, and might prefer chemotherapy plus an antiangiogenic agent for patients with right-sided mCRC [37]. Nevertheless, it is worth noting the optimal sequence with anti-EGFR mAbs in patients with *RAS* WT mCRC in the context of PTS, remains unclear. Multi-line therapy trials such as the STRATEGIC-1 phase III study (NCT01910610) would provide valuable information.

### 2.4. Rechallenge and Liquid Biopsies

**Figure 2:**
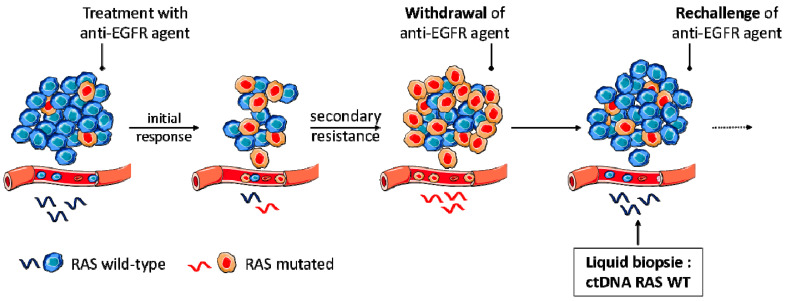
Rechallenge of anti-EGFR agents.

Resistance to targeted therapies such as anti-EGFR mAbs emerges through the selection of tumor clones harboring an innate mutation of resistance. When the anti-EGFR mAb is interrupted, the selecting pressure on the sensitive clones is withdrawn and the tumor can regain sensitivity to the targeted therapy (see Figure 2). Several strategies can be used to overcome tumor resistance (rechallenge, reintroduction, sequential therapy, dose intensification), and rechallenge seems to be the most promising in the context of anti-EGFR mAbs [38]. Rechallenge of anti-EGFR therapy could be defined as retreatment after a progression while on treatment, for a tumor that initially displayed sensitivity to the therapy [39].

Longitudinal follow-up of mutant clones seems particularly seductive for rechallenge strategies. Studies with longitudinal monitoring of ctDNA showed that *RAS* mutant clones arise in blood during anti-EGFR therapy and decline exponentially upon withdrawal of treatment, with a half-life of 4 to 5 months [40]. The CRICKET phase II study (*n* = 38) was the first prospective demonstration that a rechallenge strategy with cetuximab and irinotecan might be active in *RAS*/*BRAF* WT mCRC patients with acquired resistance to cetuximab. No *RAS* mutation was detected in blood samples from patients who experienced partial response [41,42]. Several clinical trials are currently evaluating the use of liquid biopsy in the context of anti-EGFR rechallenge (CHRONOS, NCT03227926; RASINTRO, NCT03259009) [41].

## 3. *BRAF*^V600E^ Mutation and Targeted Therapies

*BRAF*^V600E^ mutation, found in approximately 8–10% of mCRC (see Figure 1), induces am RAS-independent constitutional activation of the MAPK pathway, leading to cell proliferation and survival, and is associated with poor prognosis [43]. Although some *BRAF* mutations occur beyond the V600 hotspot, representing 22% of all *BRAF* mutations in CRC, they do not have the same biological, clinical, and therapeutic consequences as the ^V600E^ mutation [44]. These *BRAF* non-^V600E^ mutated tumors are more likely to be left-sided, with a lower grade of differentiation, and associated with better prognosis and resistance to *BRAF* inhibitors, although some might be sensitive to EGFR [45,46]. To note, fusions of the *BRAF* gene were also reported. These genetic alterations are associated with right-sided tumors and seem to confer resistance to anti-EGFR therapy [11,47,48].

*BRAF*^V600E^ CRC patients are more likely to be older, female, with right-sided tumors harboring mucinous component, associated with peritoneal and distant lymph node metastases but less pulmonary metastases [49]. Importantly about 20% of *BRAF*^V600E^ CRC exhibit the MSI phenotype, which is predictive for the efficacy of immune checkpoint inhibitors, whatever the *BRAF* mutational status (see below) [43,50].

*BRAF*^V600E^ mCRC patients have a lower probability to receive second-line treatments than the *BRAF* WT population. As a matter of fact, intensification strategies seem effective for these patients [51,52,53]. In the TRIBE study, first-line FOLFOXIRI (folinic acid, fluorouracil, oxaliplatin, and irinotecan) plus bevacizumab was associated with a non-significant improvement of overall survival for *BRAF*^V600E^ mutants, compared to FOLFIRI (folinic acid, fluorouracil, and irinotecan) plus bevacizumab (*n* = 28) [54]. Despite the small population sample of this subgroup analysis, FOLFOXIRI-bevacizumab is considered to be a valid option for chemotherapy-naïve patients with *BRAF*^V600E^ mCRC. Importantly, no survival benefit was reported for *BRAF*^V600E^ patients in the subsequent TRIBE2 phase III trial, which tested FOLFOXIRI plus bevacizumab and reintroduction, upfront after progression, versus mFOLFOX6 plus bevacizumab, followed by FOLFIRI plus bevacizumab (33 patients in each arm) [55]. The triplet chemotherapy regimen, associated with either bevacizumab or cetuximab, is currently being tested in the AIO-KRK-0116 phase II trial (NCT04034459; see Table 1).

### 3.1. Antiangiogenic Agents for the BRAF^V600E^ Mutants

There is currently no predictive biomarker for the efficacy of antiangiogenics in the overall population of mCRC patients, and the activity of these agents are not specifically proven among *BRAF*^V600E^ mCRC patients. The AVF2107g35 and AGITG MAX36 trials showed no survival improvement by adding bevacizumab to first-line IFL (bolus irinotecan, fluorouracil, and folinic acid) or capecitabine [56,57]. Yet, the RAISE study (FOLFIRI ± ramucirumab) and the VELOUR trial (FOLFIRI ± aflibercept) showed that the subset of *BRAF*^V600E^ CCR tended to benefit from these antiangiogenics in second line, although the small size of the cohorts precluded statistical significance [58,59]. All in all, these retrospective analyses suggest that antiangiogenics could be beneficial to *BRAF*^V600E^ mCRC patients, in the first-line or latter [60].

### 3.2. Anti-EGFR Agents for BRAF^V600E^ Mutants

Data regarding the efficacy of anti-EGFR therapies for *BRAF*^V600E^ patients are not clear, either as single agent or in combination with chemotherapy. Two meta-analyses were performed. A meta-analysis performed by Pietrantonio et al. suggested that anti-EGFR agents are not effective for *BRAF*^V600E^ patients [61]. However, another meta-analysis by Rowland et al. failed to detect any significant difference in the effect of anti-EGFR agents between the *BRAF*^V600E^ and *BRAF* WT population [62]. Additionally, a retrospective analysis of the *BRAF*^V600E^ subgroup, the FIRE-3 study (first-line FOLFIRI plus cetuximab versus FOLFIRI plus bevacizumab in *KRAS* WT mCRC patients) identified a higher response rate in the cetuximab arm [63]. Moreover, a recent study (VOLFI AIO KRK0109) comparing the efficacy of first line FOLFOXIRI with or without panitumumab, found a significant increase in objective response in the subgroup of *BRAF*^V600E^ patients (71% versus 22%, *n* = 14) [18]. the positive results of the BEACON study also confirm that anti-EGFR agents are valuable for the *BRAF*^V600E^ population (see below) [64]. International guidelines did not contraindicate anti-EGFR agents for *BRAF*^V600E^ mCRC patients [6,37].

### 3.3. BRAF Inhibitors

In contrast to melanoma, *BRAF* inhibitors as single agents were associated with disappointing results. One hypothesis is that *BRAF* inhibition induces feedback EGFR activation and might promote MAPK constitutive signaling. EGFR-mediated reactivation of downstream signaling pathways contributes to the inherent resistance of these tumors to *BRAF* inhibitor monotherapy [65]. With this issue in mind, several combinations of *BRAF* inhibitor, anti-EGFR agents, PI3K inhibitors, or MEK inhibitors were tested, with interesting results [66,67,68,69,70]. These studies supported the design of the phase III BEACON study, which evaluated encorafenib and cetuximab ± binimetinib versus chemotherapy (investigator choice regimen of cetuximab plus irinotecan or FOLFIRI). 665 *BRAF*^V600E^ mCRC patients with disease progression after one or two previous lines were randomized. Median OS was 9.3 months in the triplet and the doublet experimental arms, compared to 5.9 months in the control arm (HR = 0.60, 95% confidence interval 0.47–0.75 and HR = 0.61, 95% confidence interval 0.48–0.77, respectively) [64,71]. The objective response rate was statistically improved (2% in the control group, compared to 20% and 26% in the doublet and triplet group, respectively). Toxicity was manageable, with grade 3 or higher toxicities being comparable between the three arms, with gastrointestinal and skin-related events occurring in the experimental groups. A complementary quality of life analysis found a reduced risk of quality of life deterioration by more than 40% in both the doublet and the triplet group [72]. Although the study was not designed to compare the two experimental arms, the benefit-risk balance might be in favor of the encorafenib plus cetuximab combination, without the MEK inhibitor.

## 4. *RAS* Mutants and Targeted Therapies

Approximately 50% of mCRC are reported to have *KRAS*/*NRAS* pathogenic mutation. The treatment of these tumors of poor prognosis was proven to be highly challenging. They are intrinsically resistant to anti-EGFR mAbs (see above). Antiangiogenics (bevacizumab, aflibercept, ramucirumab) seem to be effective in this population but predictive biomarkers for the efficacy of these compounds are lacking [59,73,74].

*KRAS*^G12D^ (glycine 12 to aspartic acid) is the most common *KRAS* mutation in colorectal cancer. A new generation of *KRAS* inhibitor might be a game changer for this population [75]. Recently, promising results of a direct *KRAS*^G12C^ inhibitor were reported. AMG 510 is a novel small molecule that specifically and irreversibly inhibits *KRAS*^G12C^, by locking it in an inactive guanosine diphosphate (GDP)-bound state. In the first in-human study evaluating AMG 510 in adult patients with locally advanced or metastatic *KRAS*^G12C^ mutant solid tumors (CodeBreak-100; NCT03600883), ORR and DCR were 12.0% (3/25) and 80.0% (20/25), in patients with *KRAS*^G12D^-mutated mCRC [76]. Further confirmation is needed.

## 5. Microsatellite Instability and Immune Checkpoint Inhibitors

### 5.1. Colorectal Cancers, Mismatch Repair Deficiency, and Microsatellite Instability

While most CRCs develop via the chromosomal instability pathway (aneuploidy and loss of genetic material), 10–15% of them arise from the microsatellite instability (MSI) pathway. MSI is caused by the deficiency of the DNA mismatch repair (dMMR) system, resulting from a germline mutation in MMR genes (*MLH1*, *PMS2*, *MSH2*, *MSH6*) predisposing to Lynch syndrome or from an epigenetic inactivation of *MLH1* (i.e., sporadic cancers). These sporadic cases are frequently associated with the *BRAF*^V600E^ mutation [77]. MSI/dMMR is observed in approximately 10–15% of localized CRC and 4–5% of mCRC (see Figure 1) [43,78]. MSI/dMMR CRCs mainly arise from the proximal colon and display specific features such as poor differentiation, abundant tumor-infiltrating lymphocytes, and particular patterns of metastatic spread with frequent distant lymph node metastases and peritoneal involvement [49]. In localized CRC, MSI/dMMR is associated with favorable prognosis [79,80]. Data are more controversial in metastatic setting. Still, the available literature suggests MSI/dMMR mCRC are less responsive to conventional chemotherapy, compared to microsatellite stable/MMR-proficient (MSS/pMMR) tumors [43,81,82,83].

MSI/dMMR CRCs are characterized by a high tumor mutational burden (i.e., hypermutated phenotype) with highly immunogenic neoantigens arising from frameshift mutations that induce high infiltration through activated cytotoxic T CD8+ lymphocytes [84,85,86]. However, MSI/dMMR neoplasms upregulates immune checkpoints that protects MSI cancer cells from their hostile immune micro-environment [87,88].

### 5.2. Targeting the Immune System

MSI/dMMR has emerged as a major predictive biomarker for the efficacy of ICIs, especially for mCRC patients. While pMMR/MSS CRCs are mainly resistant to ICIs (i.e., cold tumors), MSI/dMMR tumors were associated with high sensitivity to immunotherapy (i.e., hot tumors). The activity of ICIs for patients with chemoresistant MSI/dMMR mCRC was demonstrated in several phase II trials, with objective response rates ranging from 33% to 58% and 12-month PFS rates between 31% and 71% [50,89,90,91,92,93,94]. Importantly, results of the non-randomized CheckMate-142 trial suggest that combinations of anti-PD1 and anti-CTLA4 mAbs might be more effective than anti-PD1 or anti-PDL1 agents alone. Pembrolizumab and nivolumab +/− ipilimumab are approved by the Food and Drug Administration, for the treatment of patients with MSI/dMMR mCRC, who progressed after fluoropyrimidine, oxaliplatin, and irinotecan-based treatment.

The efficacy of ICIs was also demonstrated as front-line treatment for patients with chemotherapy-naive MSI/dMMR mCRC. In a third cohort of the CheckMate-142 trial, 45 patients were treated with nivolumab plus ipilimumab as the first-line treatment. ORR was 77%, the 1-year PFS estimate was 77% [95]. Of major importance, the phase III KEYNOTE-177 trial recently showed pembrolizumab monotherapy to be superior to standard of care first-line chemotherapy (investigator’s choice of FOLFOX or FOLFIRI, with or without bevacizumab or cetuximab) in terms of PFS for MSI/dMMR mCRC patients. Medians of PFS were 16.5 months versus 8.2 months (HR = 0.60, 95%CI 0.45–0.80). The 12- and 24-month PFS rates were 55% and 48% with pembrolizumab versus 37% and 19% with chemotherapy. It is highly likely that pembrolizumab will become the standard of care for patients with newly diagnosed MSI/dMMR mCRC [96].

ICIs are currently evaluated for patients with localized MSI/dMMR colon cancer. The NICHE phase II study paved the way for their development in this setting and might refine therapeutic strategies for early-stage MSI/dMMR CC [97]. Indeed, in this trial testing nivolumab plus ipilimumab as neoadjuvant treatment, all 21 dMMR CC patients had a pathological response, with 95% of major responses including 12 complete pathological responses. These impressive results highlight neoadjuvant immunotherapy as a promising strategy that warrants further research. ICIs are also evaluated in combination with adjuvant chemotherapy for stage III MSI/dMMR colon cancer patients—the ATOMIC trial (NCT02912559; FOLFOX +/− atezolizumab), and the POLEM trial (NCT03827044; 24 weeks of single agent fluoropyrimidine chemotherapy or 12 weeks of oxaliplatin-based chemotherapy +/− avelumab) [28].

### 5.3. Predictive Biomarkers among MSI/dMMR CRC Patients Treated with Immunotherapy

There is currently no robust predictive biomarker for the efficacy of ICIs among MSI/MMR CRC patients. Despite the high clinical activity of ICIs in this population, some patients do not benefit from these treatments, and there is currently no clear explanation for these cases of primary resistance. It is noteworthy that misdiagnosis of MSI/dMMR status is responsible for a significant amount of cases with primary resistance to ICIs [98,99].

First, MSI/dMMR tumors harboring the *BRAF*^V600E^ mutation seem to be quite sensitive to MSI/dMMR *BRAF* WT diseases [50]. Second, PD-1 expression, beta-2-microglobulin mutations, and major histocompatibility complex class I expression were not found to be associated with resistance to ICI [50,90,100]. Loss-of-function mutations in Janus kinases JAK1/2 might lead to primary resistance of MSI/dMMR mCRC to ICI [101]. Interestingly, the tumor mutational load was reported to predict ICI efficacy in 2 small cohort studies (<33 patients) [102,103]. Data are also emerging for the predictive impact of the immune infiltrate for the efficacy of ICIs. Indeed, in a recent work by Loupakis and colleagues, the level of T cell infiltration was linked to better response, PFS, and OS [98]. All these results deserve confirmation in larger prospective studies.

## 6. *HER2* and Anti-HER2 Agents

Approximately 1% to 8% of CRC harbor *HER2* gene amplification (see Figure 1) [104,105,106,107]. HER2 overexpression is associated with distal cancers, with a frequency of 4.3–5.4% for rectal cancers, and *KRAS* WT status, although these 2 genetic alterations could be observed together [106,108,109]. While the prognostic significance of *HER2* amplifications is controversial, many arguments are accumulating in favor of a negative predictive value of *HER2* amplifications for anti-EGFRs efficacy (ORR, PFS, OS) [11,110,111].

The diagnostic method for HER2 testing in CRC was standardized by the Heracles diagnostic criteria, with first-line immunohistochemistry (IHC) analysis followed, if appropriate, by FISH analysis (fluorescence in situ hybridization). Positivity is defined by an IHC 3+ score or an IHC 2+ score associated with FISH positivity [112].

Evidence is growing for the efficacy of anti-HER2 agents for patients with *HER2*-positive mCRC. Combinations of trastuzumab plus lapatinib, trastuzumab with pertuzumab, and trastuzumab plus tucatinib were tested in phase II studies (Heracles-A, MyPathway, and Mountaneer, respectively). They showed promising response rates of 30 and 32, and a 55% and median PFS of 4.7, 2.9, and 6.2 months, respectively [109,113,114]. Patients with *HER2*-positive and *KRAS*-mutated mCRC were excluded from the Mountaneer and Heracles-A trials, but it is worthy to note that one *HER2*-positive and *KRAS*-mutated mCRC patient experienced an objective response in the MyPathway study [109,113,114]. In contrast, the Heracles-B trial, with the association of pertuzumab and trastuzumab emtansine, failed to reach its primary endpoint (ORR), even if the median PFS was 4.7 months [115]. Recent report of the DESTINY-CRC01 phase II trial has brought to light trastuzumab-deruxtecan as a potential game changer. Fifty patients with chemoresistant HER2-positive mCRC were treated with this antibody-drug conjugate (composed of an anti-HER2 antibody and topoisomerase I inhibitor). The confirmed objective response rate was 45% (24/53). Even patients with prior anti-HER2 agents derived benefit from this treatment, with an objective response rate of 43.8% (7/16 pts). Two deaths from drug-related interstitial lung disease were reported.

Overall, anti-HER2 agents are highly seductive therapies for the *HER2*-positive population, but data from randomized trials are lacking in rigorous evaluation of their added value. The only ongoing randomized study is a phase II trial comparing trastuzumab and pertuzumab to irinotecan and cetuximab, in patients with *HER2*-positive *RAS*/*RAF* WT mCRC (NCT03365882).

## 7. *NTRK* Gene Fusions and TRK Inhibitors

*NTRK* gene fusions recently emerged as a highly seductive therapeutic target for cancer patients. TRK inhibitors (larotrectinib, entrectinib) demonstrated an impressive clinical activity in this population, regardless of histological type. In single-arm studies, larotrectinib showed an objective response rate of 75%, with a time of response superior to 6 months in 73% of cases, and entrectinib, an ORR of 57%, with a time of response superior to 6 months in 68% of cases [116,117]. These results led to accelerated approvals by the FDA for the treatment of refractory solid tumors with the *NTRK* gene fusion, whatever the tumor type.

Strategies for *NTRK* fusion screening are based on immunohistochemistry fluorescence in situ hybridization, RT-PCR (reverse transcription polymerase chain reaction) and next-generation sequencing, depending on the probability of *NTRK* fusion [118,119]. *NTRK* fusions are rare in colorectal cancers, with an incidence of 0.23–0.97% (see Figure 1) [48,120,121,122,123]. It is, therefore, necessary to select the population to be screened.

Characteristics of *NTRK* fusion-positive CRC patients are female, right-sided primary tumor location, *RAS*/*RAF* WT status, and MSI phenotype [121]. Intriguingly, the MSI phenotype was consistently reported in association with *NTRK* fusions. More precisely, these genetic alterations seemed to occur in the context of *BRAF* WT tumors with hypermethylation of the *MLH1* gene promoter [124]. The incidence of *NTRK* fusions was estimated at approximately 42% in this molecularly selected population [48]. There is currently no data about the efficacy of *NTRK* inhibitors or immune checkpoint inhibitors in this specific biological entity.

## 8. Conclusions

Substantial advances were made in the individualization of therapeutic strategies for mCRC patients over the last 10 years. Responders to anti-EGFR therapies can be specifically selected with an enlarged panel of biomarker, and the therapeutic strategies can be optimized with the longitudinal follow-up of ctDNA. Patients with *BRAF*^V600E^ mCRC, who were long left behind, now have effective therapeutic options. Beyond highly seductive but quite rare targets, such as *HER2* amplification and *NTRK* fusions, the most striking revolution for targeted therapies in CRC patients comes from ICIs that were a breakthrough for patients with MSI/dMMR tumors. Patients’ outcomes were dramatically improved, and this enforced clinicians and researchers to conceptualize CRC as at least 2 distinct diseases—the MSI/dMMR tumors, and the others (Table 2). Importantly, the development of ICIs is associated with methodological issues in relation with the pseudoprogression phenomenon and long-term survivals. This observation highlights the necessity to develop new study designs and to anticipate such issues in pre-planned statistical analyses.

This evolution brings to light the international CMS classification, that clustered CRC into 4 distinct consensus molecular subtypes (CMS): CMS1 (MSI Immune), hypermutated, microsatellite unstable, *BRAF*^V600E^ mutated, with strong immune activation; CMS2 (Canonical), epithelial, chromosomally unstable, with marked WNT and MYC signaling activation; CMS3 (Metabolic), epithelial, with evident metabolic dysregulation; and CMS4 (Mesenchymal), prominent TGF-β activation, stromal invasion, and angiogenesis. The CMS classification might help identify new pathways to target, such as the TGF-β pathway or others. Although it is currently used in research investigation, the role of CMS classification for therapeutic strategies remains to be defined, and its feasibility in routine practice to be demonstrated.

To conclude, translational research seems more necessary than ever to understand the biological specificities of these various types of CRC, the innate and acquired mechanisms of resistance to targeted therapies, to bring progress to all patients.

## Figures and Tables

**Table 1:** Ongoing clinical trials for patients with *BRAF*^V600E^ metastatic colorectal cancer.

Therapy	Phase	Condition	Primary Endpoint	NCT Identifier
Encorafenib ^1^ + cetuximab ^2^ + nivolumab ^4^	1/2	2 or 3rd line	ORR, DLT	NCT04017650
Encorafenib ^1^ + binimetinib ^3^ + nivolumab ^4^	1/2	>1st line	ORR, DLT	NCT04044430
Dabrafenib ^1^ + trametinib ^3^ + PDR 001 ^4^	2	Any line	ORR, DLT	NCT03668431
FOLFOXIRI + cetuximab ^2^ or bevacizumab ^5^	2	1st line	ORR	NCT04034459
FOLFIRI + cetuximab ^2^ + vemurafenib ^1^	2	-	ORR	NCT03727763
Irinotecan + AZD 1775 ^6^	1	>1st line	DLT	NCT02906059
Panitumumab ^2^ + trametinib ^3^	2	>2nd line	ORR	NCT03087071

^1^ RAF inhibitor; ^2^ EGFR inhibitor; ^3^ MEK inhibitor, ^4^ anti PD(L)-1; ^5^ Anti VEGF; ^6^ Wee-1 inhibitor; ORR: objective response rate; and DLT: dose limiting toxicities.

**Table 2:** Molecular subtypes of colorectal cancer and targeted treatment options.

Molecular Subtypes	Targeted Therapies
MSI, whatever the *RAS*/*RAF* mutational status	Immune checkpoint inhibitor(s)
*RAS*/*RAF* wild-type	Anti-EGRF mAbs
*BRAF*^V600E^ mutated	Encorafenib + cetuximab +/− binimetinib
*RAS* mutated	No current targeted therapy, ongoing trials with new-generation KRAS inhibitors
*HER2* amplified/mutated	Anti-HER2 mAbs/inhibitors (trastuzumab, pertuzumab, lapatinib), anti-HER2 antibody-drug conjugate (trastuzuab deruxtecan)
*NTRK* fusion-positive	TRK inhibitor (Larotrectinib, entrectinib)
